# Heat of Formation of Calcium Aluminate Monocarbonate at 25 °C

**DOI:** 10.6028/jres.065A.023

**Published:** 1961-06-01

**Authors:** H. A. Berman, E. S. Newman

## Abstract

The heat of formation of 3CaO·Al_2_O_3_·CaCO_3_·10.68H_2_O at 25 °C was determined by the heat-of-solution method, with 2*N* HCl as the solvent and 3CaO·Al_2_O_3_·6H_2_O and CaCO_3_ as the reactants. The heat of solution of CaCO_3_, to form dissolved CO_2_, was obtained by a new technique and a modified calculation which served to include the heat of vaporization of the gas escaping in the reaction and resulted in a higher value than those obtained by Wells and Tayıor and by Bäckström in determinations representing only partial solution of CO_2_. The results obtained were:
*kj/mole**kcal/mole*3CaO·Al_2_O_3_·CaCO_3_·10.68H_2_O Heat of formation  from elements, 
$\Delta H_{f}^{{}^{\circ}}$−1956     from reactants and H_2_O−18.9 Heat of solution in 2*N* HCl−532.9−127.43CaO·Al_2_O_3_·6H_2_O Heat of solution in 2*N* HCl−576.6−137.8CaCO_3_ Heat of solution in 2*N* HCl−35.0−8.4

## 1. Introduction

Hydrated calcium aluminate monocarbonate, 3CaO·Al_2_O_3_·CaCO_3_·11H_2_O, is one member of a series of complex salts represented by the general formula 3CaO·Al_2_O_3_·Ca*X*·*n*H_2_O, in which *X* is a divalent anion or two units of a monovalent anion, and *n* is 10 to 12. Turriziani and Schippa [1]^1^ have observed and identified the monocarbonate in a film which formed on the surface of water in which cubes of aluminous cement paste were stored. The limited possibility of its formation in setting mortar and concrete is indicated by its stability only at CO_2_ pressures below the partial pressure of CO_2_ in air [2]. It has nevertheless been observed in test borings from portland cement concrete road surfaces [3].

As part of a continuing investigation of the thermochemical properties of substances occurring in hydraulic cements and their reaction products, the heat of formation of hydrated calcium aluminate monocarbonate has been determined.

It should be noted that the formula 3CaO·Al_2_O_3_·CaCO_3_·11H_2_O is an idealized formula in which the number of moles of H_2_O has been rounded off. This compound, as with many similar compounds encountered in portland cement chemistry, does not always have a definite water content. The actual water content in the samples prepared varied from 10.58 to 10.88. The average figure of 10.68H_2_O was used in the calculations.

Measurements were made of the heat evolved at 25 °C in the reaction

$$3\text{CaO}\cdot {\text{Al}}_{2}O_{3}\cdot 6H_{2}O+{\text{CaCO}}_{3}+4.68H_{2}O\overset{\Delta H_{1}}{\to }3\text{CaO}\cdot {\text{Al}}_{2}O_{3}\cdot {\text{CaCO}}_{3}\cdot 10.68H_{2}O\;$$ (1)

The heat of this reaction is the difference between the sum of the heats of solution of the reactants and the heat of solution of the product, in accordance with the following equations:

$$3\text{CaO}\cdot {\text{Al}}_{2}O_{3}\cdot 6H_{2}O+12\text{HCl}\overset{\Delta H_{2}}{\to }[3{\text{CaCl}}_{2}+2{\text{AlCl}}_{3}+12H_{2}O]\;$$ (2)

$${\text{CaCO}}_{3}+[3{\text{CaCl}}_{2}+2{\text{AlCl}}_{3}+12H_{2}O]+2\text{HCl}\overset{\Delta H_{3}}{\to }[4{\text{CaCl}}_{2}+2{\text{AlCl}}_{3}+13H_{2}O]+{\text{CO}}_{2}$$ (3)

$$\left[4{\text{CaCl}}_{2}+2{\text{AlCl}}_{3}+13H_{2}O]+4.68H_{2}O\overset{\Delta H_{4}}{\to }[4{\text{CaCl}}_{2}+2{\text{AlCl}}_{3}+17.68H_{2}O\right]$$ (4)

$$[4{\text{CaCl}}_{2}+2{\text{AlCl}}_{3}+17.68H_{2}O]+{\text{CO}}_{2}\overset{\Delta H_{5}}{\leftarrow }3\text{CaO}\cdot {\text{Al}}_{2}O_{3}\cdot {\text{CaCO}}_{3}\cdot 10.68H_{2}O+14\text{HCl}$$ (5)

Summation:

$$\begin{array}{c} 3\text{CaO}\cdot {\text{Al}}_{2}O_{3}\cdot 6H_{2}O+{\text{CaCO}}_{3}+4.68H_{2}O\overset{\Delta H_{1}}{\to }3\text{CaO}\cdot {\text{Al}}_{2}O_{3}\cdot {\text{CaCO}}_{3}\cdot 10.68H_{2}O\\ \Delta H_{1}=\Delta H_{2}+\Delta H_{3}+\Delta H_{4}+\Delta H_{5} \end{array}$$ (1)

The heat of solution of each of the reactants and of the product of this reaction was measured in HCl·26.61H_2_O (2.00*N* HCl at 25 °C). The heats of formation of the reactants were taken from Circular 500 [4] of the National Bureau of Standards.

All calculations in this paper are based on the 1957 atomic weight table, and on the thermochemical calorie, defined as exactly 4.184 absolute joules.

## 2. Materials, Apparatus, and Procedure

### 2.1. Preparation of 3CaO·Al_2_O_3_·CaCO_3_·11H_2_O

Three samples, two of which closely approached the stoichiometric composition 3CaO·Al_2_O_3_·CaCO_3_·11H_2_O, were prepared and studied. Their properties and details of their preparation are listed in table 1. They were obtained by mixing solutions of calcium aluminate, calcium hydroxide (prepared from reagent calcium carbonate), and reagent sodium carbonate, according to the method of Turriziani and Schippa [1]. The calcium aluminate solution was prepared by shaking aluminous cement with distilled water for periods up to 3 hr. The solution, containing up to 2.0 g Al_2_O_3_ and 1.2 g CaO per liter, is metastable and becomes cloudy soon after its maximum concentration is reached. It was filtered quickly and the filtrate used immediately to prepare the monocarbonate compound. A sample of the filtrate was taken at the same time to determine the concentration of CaO and Al_2_O_3_. Eight to nine liters of saturated Ca(OH)_2_ solution were added to 2 liters of the calcium aluminate solution, the mixture stirred a few seconds, after which 3.50 to 3.75 g Na_2_CO_3_ in 250 ml of water was added slowly with continued stirring. After the mixture had been allowed to stand about 2 months, the precipitate was filtered off and dried over saturated MgCl_2_ solution (33% relative humidity) until the ignition loss was constant (requiring about 2 months).

Precautions were taken to exclude atmospheric CO_2_ during every step of the process including preparation of the reagents, mixing, filtration, and drying. These precautions are described in another publication [5].

X-ray diffraction patterns of the samples were obtained by the powder method on a Geiger-counter diffractometer with copper K*α* radiation. The patterns, one of which has been published [2], agree closely with the original pattern of Turriziani and Schippa [1].

The oxide composition of the samples was obtained by chemical analysis. The designation and calculation of the amounts of compounds present as impurities was based on the chemical analysis and optical microscopic examination.

### 2.2. Preparation of Hydrated Tricalcium Aluminate

Tricalcium aluminate hydrate, 3CaO·Al_2_O_3_·6H_2_O, was prepared by autoclaving 3CaO·Al_2_O_3_ at 150 °C for several days, according to the method of Thorvaldson, Brown, and Peaker [6]. The hydrated aluminate was dried over potassium hydroxide. Chemical analysis gave the composition 3CaO·Al_2_O_3_·5.859H_2_O (plus 0.007 mole CaCO_3_ per mole as impurity). The anhydrous aluminate had been prepared by repeated heating of lime and alumina with intervening moistening of the material to aid in dispersing the CaO, as described by the same authors.

### 2.3. Preparation of the Calcium Carbonate

Low-alkali reagent calcium carbonate was used. The reagent was moistened with 3 percent of its weight of distilled water, pelleted at 4,000 psi, and dried at 110 °C to constant weight. This treatment served to agglomerate the fine particles and decrease (but not completely eliminate) the tendency of the CaCO_3_ to adhere to the glass funnel through which samples were normally introduced into the calorimeter.

The CaCO_3_ was largely calcite. Since the difference between the heats of formation of calcite and aragonite [4] is smaller than the reproducibility of the calorimetric measurements, the presence of aragonite in the CaCO_3_ would have no effect on the results of the heat-of-solution determinations.

### 2.4. Heat-of-Solution Measurements

**Figure 1:**
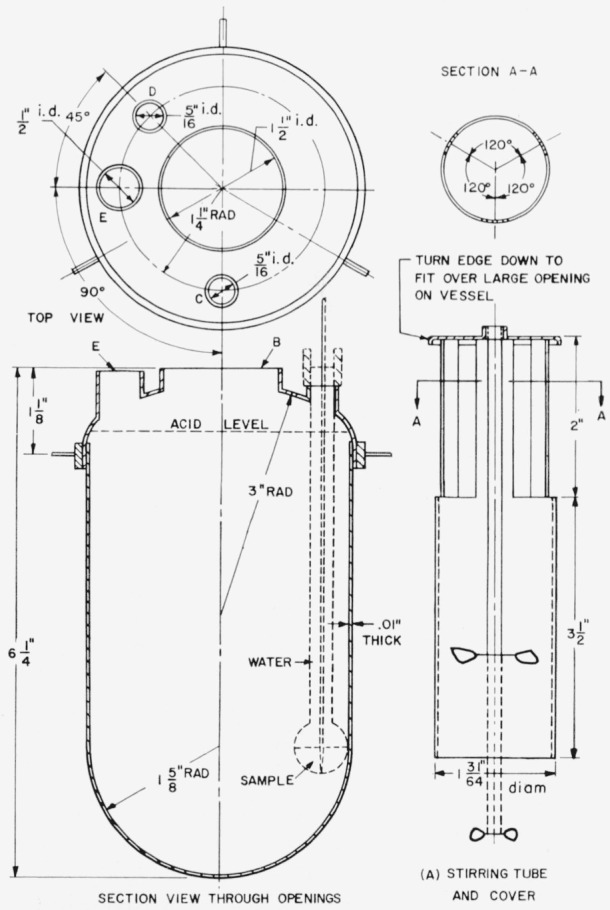
Improved heat-of-solution calorimeter The glass bulb shown with dashed lines was used for determinations of the heat of solution of CaCO_3_. Opening C normally contains a funnel instead of a glass bulb.

The heats of solution of the compounds were determined in HCl·26.61_4_H_2_O (2.00*N* HCl at 25 °C). A precision calorimeter, described by Newman [7], was used for determinations on all the materials except the CaCO_3_. For this compound an improved calorimeter was used, which is shown in figure 1. The major advantage of the newer calorimeter is its one-piece design, except for the stirring tube and stirrer (A) which fit over and close a 1.5-in. dıam opening (B) in the upper surface. Two stirrers can be used, a 4-in. stirrer with a 4-blade propeller, and a 6¼-in. stirrer (shown with dashed line in the figure) with an additional 2-blade propeller at the end. The 4-in., single-propeller, stirrer was used for the heat of solution of CaCO_3_. Thermal equilibrium in the calorimeter is reached much sooner than in the older design, which featured a heavy cover and large temperature lags. The sample is introduced through opening C, either by means of a funnel or in a glass bulb as indicated by the dashed lines in the drawing. The glass bulb was used with CaCO_3_, as described in section 2.5. The entire calorimeter assembly, except for the bulb or funnel and for the outer supporting ring, is made of platinum. The calorimeter otherwise is similar to the earlier design. Opening D is fitted with an electric heater for calibration purposes and for setting the initial temperature range, and opening E contains the resistance thermometer. The calorimeter is placed inside an air jacket and the jacket-calorimeter assembly is submerged in a constant-temperature water bath controlled to ±0.005 °C.

Since it was inconvenient to prepare the calcium aluminate monocarbonate in large quantities, 1–g samples of the compound were used with the normal quantity of acid (600 g) for the older calorimeter. With the newer calorimeter, 0.2–g samples of CaCO_3_ were used with 740 g of acid (corresponding to 0.18 g in 600 g acid), and 0.7 g of hydrated tricalcium aluminate was added to the acid before the addition of CaCO_3_. These quantities are required by the stoichiometry of eqs (1) and (3).

In the determination of the heat of solution of 3CaO·Al_2_O_3_·6H_2_O, the sample size was 0.2 to 0.4 g. These determinations had been made previously to calculate the heat of formation of calcium aluminate trisulfate (calcium trisulfoaluminate) [5]. In accordance with eq (1), the proper sample size should be 0.67 g. However, the value obtained for the smaller samples (370.4 cal/g) is not inconsistent with values obtained by Thorvaldson, Brown, and Peaker [6] from determinations in which 2.9–g samples were used. When their results are adjusted to allow for the use of HCl·26.6H_2_O instead of HCl·20H_2_O, thermo chemical calories (4.1840 abs j/cal) instead of 20° calories (4.181 abs j/cal), and a final temperature of 25 °C instead of 20°, the result is 373.1 cal/g. The difference, 2.7 cal/g, or 1.0 kcal/mole, is in the wrong direction to be attributed to differences in sample size. There is thus no measurable dilution effect due to variations in sample size. The value obtained with the smaller samples was therefore used.

The heat effect of adding the 4.68 moles of water appearing in eqs (1) and (4) was estimated as the partial molal heat content of water in 2*N* HCl [4], neglecting the contribution of the small amounts of other solutes present.

### 2.5. Heat of Solution of the CaCO_3_

The determination of the heat of solution of CaCO_3_ presents problems because of the evolution of CO_2_ when the sample is dissolved in acid. Two reactions take place:

$${\text{CaCO}}_{3}\;(c)+2\text{HCl}\;(\text{aq})\underset{\left(\text{exothermic}\right)}{\to }{\text{CaCl}}_{2}\;(\text{aq})+H_{2}O\;(l)+{\text{CO}}_{2}\;(\text{aq})$$ (6)

$${\text{CO}}_{2}\;(\text{aq})\underset{\left(\text{ecdothermic}\right)}{\to }{\text{CO}}_{2}\;(g)$$ (7)

The problem is twofold: (a) the evolution of CO_2_ from solution is endothermic; an uncertain amount of heat is lost depending on how much CO_2_ escapes. (b) The CO_2_ gas carries with it some of the sensible heat produced by equation (6) if it is not in complete thermal equilibrium with the solution before it is evolved. The sensible and latent heat of water vapor in the CO_2_ gas is lost as well. All these effects produce low results for the heat of solution and an inaccurate final rating period for the calculation of thermal leakage.

Various investigators have attacked the first difficulty by determining the heat of solution of CaCO_3_ both in HCl saturated with CO_2_ and in HCl not saturated with CO_2_. A smaller heat of solution is obtained in the first solvent because all the CO_2_ escapes. The second solvent retains some of the CO_2_ and gives a larger value. Working with 1.5-g samples in 640 g of CO_2_-saturated *2N* HCl, Wells and Taylor [8] obtained –Δ*H* = 59.2 cal/g CaO or 33.2 cal/g CaCO_3_ (3.32 kcal/mole) at 25 °C. Working with 10-g samples in 800 g of CO_2_-saturated *N* HCl at the same temperature, Bäckström obtained 3.26 kcal/mole [9, 10].

The agreement between these two investigators was not as good when the HCl was not saturated with CO_2_, principally because the sample and acid weights were different, resulting in different ratios of CO_2_ dissolved in the acid to CO_2_ escaping from the calorimeter in each case. Wells and Taylor, using 2*N* HCl at 25 °C and the same relative weights as for CO_2_-saturated HCl, obtained a heat of solution of *–*Δ*H* = 105.0 cal/g CaO or 58.9 cal/g CaCO_3_ (5.89 kcal/mole). Bäckström, using *N* HCl at 25 °C and the same relative weights he had used before, obtained 4.50 kcal/mole.

The heats of solution of the aluminate monocarbonate and of the tricalcium aluminate had been determined in 2*N* HCl not saturated with CO_2_. It was therefore necessary to determine the heat of solution of the CaCO_3_ in the same acid and to adopt a sample size that would result in the same concentration of CO_2_ as for the aluminate carbonate. A CaCO_3_ sample of 0.2 g does not release enough CO_2_ to saturate 740 g of 2*N* HCl at 25 °C. (The solubility of CO_2_ in 2*N* HCl at 25 °C is 0.8 g/liter CO_2_ in the liquid for each g/liter in the gas phase or 1.6 g/liter at 760 mm pressure [11].) Therefore, it should be expected that all the CO_2_ would dissolve, thus producing the maximum heat of solution, that is, the heat effect of eq (6). However, CO_2_ determinations made on the calorimeter acid before and after the calorimetric determinations showed that only about two-thirds of the available CO_2_ dissolved completely even though saturation was not attainable. Presumably this is due to the fact that chemical equilibrium was not established owing to the rapid solution of the sample.

It is difficult to introduce powdered CaCO_3_ into a calorimeter through a funnel because it adheres to the glass and forms lumps at the bottom of the funnel stem, even after the pressure treatment described in section 2.3. It was therefore introduced in a glass bulb (see fig. 1) placed in the acid before assembling the calorimeter. After an initial rating period to determine the stirring energy of the calorimeter, the bulb was broken by a glass rod actuated by a 10-g weight falling through a distance of 69 cm. The temperature of the calorimeter was read at 1-min intervals until the rise was steady, then at 2-min intervals for a total of about 1 hr. Corrections were made to the observed temperature rises by subtracting the stirring energy and the thermal leakage between the bath and the calorimeter. The thermal-leakage constant had been determined previously in the calorimeter calibration.

**Figure 2:**
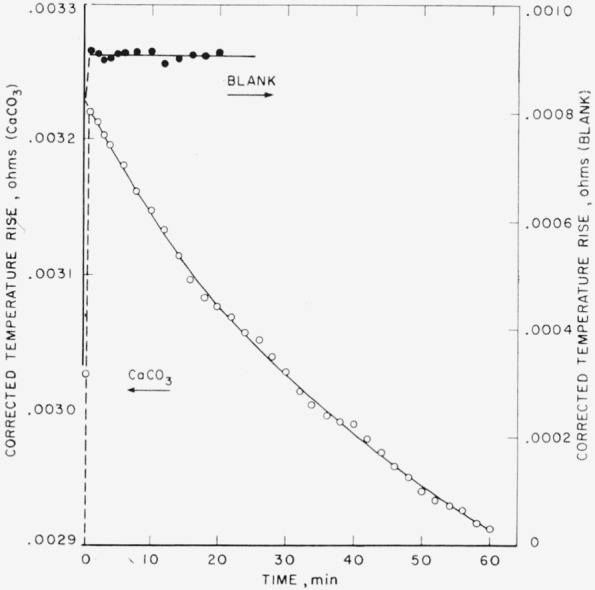
The corrected temperature rise of a 0.2247–g sample of CaCO_3_ plotted against the time after the sample was introduced into the calorimeter The curves for both blank and sample start from the origin, representing zero temperature rise at zero time. Open circles and ordinates on left refer to the CaCO_3_. Closed circles and ordinates on right refer to a blank run. The sample was placed in a glass bulb and covered with water. The blank contained water alone.

The corrected rises obtained in this manner for a typical calorimeter run are plotted against time (open circles) in figure 2. The corrected temperature rise for CaCO_3_ increases rapidly at the start and reaches a maximum (about 95% of the rise occurs in the first half-minute). It then falls as CO_2_ is evolved, approaching a constant value. A first-order decay law

$$d\theta /dt=-c(\theta -\theta_{f})$$ (8)

fits the descending curve quite well; *θ* represents the corrected temperature rise at any time *t, θ_f_* the final temperature rise. From this relationship it is possible to calculate *θ*_0_, the corrected temperature rise at zero time when no CO_2_ has escaped. This rise, multiplied by the heat capacity of the calorimeter, is taken as the true heat of solution of CaCO_3_ in 2*N* HCl in accordance with eq (6).

The tendency of sensible heat to escape with the CO_2_ was reduced by covering the sample of CaCO_3_ in the bulb with a few milliliters of distilled water. Any bubbles rising in the bulb stem give up their sensible heat to the water. At the same time, the water pushes the sample directly into the calorimeter acid, causing most of the bubbles to escape through the acid surrounding the bulb. Thermal equilibrium is thereby facilitated. Blank runs were made to determine the energy of dilution of the acid with the small quantity of water. Figure 2 (closed circles) shows the corrected rise for the blank run plotted against the time after introduction of the sample.

## 3. Results and Discussion

### 3.1. Heats of Solution of the Preparations

The heats of solution obtained on the preparations of calcium aluminate monocarbonate were corrected for the small quantities of impurities present. The heats of solution of the preparations and the corrected heats of solution of the pure monocarbonates themselves are summarized in table 1.

The heats of solution of the reactants, 3CaO·Al_2_O_3_·5.859H_2_O and CaCO_3_, are shown in table 2.

The heat capacities obtained in the calorimeter calibrations are listed in tables 3 (a and b).

Additional data from the heat-of-solution determinations are shown in table 4 and 5.

### 3.2. Correction for Impurities

The calculation of the composition of a sample and of the heat of solution of the pure aluminate carbonate present in a sample is shown in the appendix, with sample 1 as the example.

The spread for the heat of solution of the three preparations is 4.51 cal/g before correction for impurities. After correction, the spread is 1.73 cal/g. The average corrected heat of solution of the three samples is –Δ*H*=226.36±0.56^2^ cal/g or 127.37±0.38 kcal/mole. The average water content of the pure compound in the 3 samples is 10.68H_2_O.

### 3.3. Heat of Solution of the CaCO_3_

The heat of solution eventually determined for the CaCO_3_ was higher (–Δ*H*=83.6 cal/g) than the values of Wells and Taylor (58.9 cal/g) [8] and Bäckström (44.9 cal/g) [10], obtained by these authors with partial evolution of CO_2_. The accuracy of the higher figure obtained in this work may be checked in two different ways.

The heat of the reaction

$$\text{CaO}\;(c)+{\text{CO}}_{2}\;(\text{aq})\overset{\Delta H_{9}}{\to }{\text{CaCO}}_{3}\;(c)$$ (9)

may be calculated by several methods, one of which requires the heat of solution of CaCO_3_ which is to be checked:

- From the heats of formation of the three compounds in equation 9 [4], Δ*H*_9_=—37.9 kcal/mole.
- From the heat of the same reaction, but with CO_2_ (g) as a reactant instead, and from the heat of vaporization of CO_2_ from solution, both values obtained from Bäckström’s work [10, 12]:
CaO (c) +CO_2_ (g) → CaCO_3_ (c)Δ*H*_10_ = −42.6   CO_2_ (aq) → CO_2_ (g)Δ*H*_11_ = + 4.9
Δ*H*_9_ = −37.7
  If the heat-of-formation figures for CO_2_ from NBS Circular 500 [4] are used, Δ*H*_11_ is calculated to be +4.64. If this figure is added to Bäckström’s value for Δ*H*_10_, Δ*H*_9_ becomes −38.0.
- From the heats of solution of CaO and CaCO_3_ in 2*N* HCl, based on Wells and Taylor’s value for CaO of −46.7 kcal/mole. [8] and the heat of solution of CaCO_3_ obtained in this work, Δ*H*_9_=−38.3 kcal/mole.

A second check on the value reported here may be made by calculating the heat of solution of CaCO_3_ in 2*N* HCl not saturated with CO_2_ from the heat of solution in CO_2_-saturated acid and from the heat of vaporization of CO_2_ from solution (Δ*H*_11_). To Wells and Taylor’s value of −3.32 [8] or Bäckström’s value of −3.26 [10] for the heat of solution may be added −4.9 (Bäckström) or −4.64 (NBS Circ. 500) [4] to obtain values ranging from −7.9 to −8.2 kcal/mole for the heat of solution in 2*N* HCl not CO_2_-saturated. This compares with −8.4 kcal/mole determined in this work.

There may still be a residual error in this determination. The corrected temperature rise extrapolated analytically to zero time represents the correct enthalpy change for equation 6 only if all the CO_2_ has dissolved at the start. Some of the CO_2_ may, however, be evolved as a gas almost immediately without preliminary solution. The result obtained by this technique may therefore still be somewhat low. An error in the opposite direction results from the fact that the solution of the CaCO_3_ takes place in finite time so that the extrapolation should really be made to some point between zero time and 1 min.

The heat of solution of 3CaO·Al_2_O_3_·CaCO_3_·*n*H_2_O was not materially different when determined by ordinary methods or by the technique used for CaCO_3_. This is attributed to the fact that the aluminate monocarbonate is slower to react with acid, and the CO_2_ is released more slowly. Chemical equilibrium is presumably obtained more easily, with complete solution of the CO_2_. The difference in activity is further evidence that CO_2_ is bound differently in the monocarbonate than in CaCO_3_, as concluded from differential thermal analysis and infrared patterns by Carlson and Berman [2].

### 3.4. Heat of Formation of the Product from the Reactants

The heat of the reaction

$$3\text{CaO}\cdot {\text{Al}}_{2}O_{3}\cdot 5.859H_{2}O\;(c)+{\text{CaCO}}_{3}\;(c)+4.82H_{2}O\;(l)\to 3\text{CaO}\cdot {\text{Al}}_{2}O_{3}\cdot {\text{CaCO}}_{3}\cdot 10.68H_{2}O\;(c)$$ (10)

is calculated from the heats of solution of the reactants and products as follows:

$$\begin{array}{l} 3\text{CaO}\cdot {\text{Al}}_{2}O_{3}\cdot 5.859H_{2}O\;(c)+654.375\;(\text{HCl}\cdot 26.614H_{2}O)\;(\text{aq})\to [3{\text{CaCl}}_{2}+2{A1C1}_{3}+\\ 642.375\;(\text{HCl}\cdot 27.130H_{2}O)]\;(\text{aq});\Delta H=\;+139.16\;\text{kcal}. \end{array}$$ (11)

$$\begin{array}{l} {\text{CaCO}}_{3}\;(c)+[642.375\;(\text{HCl}\cdot 27.130H_{2}O)+3{\text{CaCl}}_{2}+2{A1C1}_{3}]\;(\text{aq})\to {\text{CO}}_{2}(\text{aq})+[4{\text{CaCl}}_{2}\\ +2{A1C1}_{3}+640.375\;(\text{HCl}\cdot 27.216H_{2}O)]\;(\text{aq});-\Delta H=+8.37\;\text{kcal}. \end{array}$$ (12)

$$\begin{array}{l} {\text{CO}}_{2}\;(\text{aq})+[4{\text{CaCl}}_{2}+2{A1C1}_{3}+640.375\;(\text{HCl}\cdot 27.223H_{2}O)]\leftarrow 3\text{CaO}\cdot {\text{Al}}_{2}O_{3}\cdot {\text{CaCO}}_{3}\cdot 10.68H_{2}\text{O}\;(\text{c})\\ +654.375\;(\text{HCl}\cdot 26.614H_{2}O)\;(\text{aq});-\Delta H\;=-127.37\;\text{kcal}. \end{array}$$ (13)

$$640.375(\text{HCl}\cdot 27.216H_{2}O)\;(\text{aq})+4.82H_{2}O(1)\to 640.375\;(\text{HCl}\cdot 27.223H_{2}O)(\text{aq});-\Delta H=+0.07\;\text{kcal}.$$ (14)

The heat of reaction (eq (10)) is the sum of the heat effects of eqs (11), (12), (13), and (14), or *—*Δ*H* = 20.23 kcal/mole 3CaO·Al_2_O_3_·CaCO_3_·10.68H_2_O.

### 3.5. Correction for the Water Content of the Tricalcium Aluminate

This correction is found by adding to the heat of reaction for equation 10 the heat of the following reaction :

$$3\text{CaO}\cdot {\text{Al}}_{2}O_{3}\cdot 6H_{2}O\to 3\text{CaO}\cdot {\text{Al}}_{2}O_{3}\cdot 5.859H_{2}O+0.141H_{2}O.$$ (15)

This correction has been estimated previously as −Δ*H*=−1.36 kcal/mole 3CaO·Al_2_O_3_ [5]. The heat of reaction is therefore −Δ*H*=20.23 −1.36 = 18.87 kcal, for the equation

$$3\text{CaO}\cdot {\text{Al}}_{2}O_{3}\cdot 6H_{2}O+{\text{CaCO}}_{3}+4.68H_{2}O\to 3\text{CaO}\cdot {\text{Al}}_{2}O_{3}\cdot {\text{CaCO}}_{3}\cdot 10.68H_{2}O.$$ (16)

### 3.6. Heat of Formation

The heat of formation of the calcium aluminate monocarbonate is the sum of the heat effect of equation 16 and the heats of formation of the reactants in that equation [4], and is calculated as follows:

$\Delta H_{f}^{{}^{\circ}}$ 3CaO·Al_2_O_3_·6H_2_O= −1329
$\Delta H_{f}^{{}^{\circ}}$ CaCO_3_= −288.45
$\Delta H_{f}^{{}^{\circ}}$ 4.68H_2_O= −319.72Δ*H*3CaO·Al_2_O_3_·CaCO_3_·10.68H_2_O from these reactants= −18.87

$\Delta H_{f}^{{}^{\circ}}$ 3CaO·Al_2_O_3_·CaCO_3_·10.68H_2_O= −1956 kcal/mole

The calculated uncertainty of this result, based on a standard deviation for the heat of solution of 0.38 kcal/mole for the aluminate carbonate, 0.12 for the tricalcium aluminate hydrate, and 0.05 for the calcium carbonate, is 0.40 kcal/mole. This value does not include the uncertainty in the heat of formation of the reactants, which is certainly of the order of 1 kcal/mole for the hydrated tricalcium aluminate, nor the uncertainties due to chemical analysis.

## 4. Summary

Determinations were made of the heat of solution of 3CaO·Al_2_O_3_·CaCO_3_·10.68H_2_O in 2.00*N* HCl at 25 °C. Calculations were made of the heat of formation of this compound from 3CaO·Al_2_O_3_·6H_2_O, CaCO_3_, and H_2_O; and from the elements. A new technique was employed to determine the heat of solution of CaCO_3_, in which most of the sensible heat lost by escaping CO_2_ gas was retained by improved thermal equilibrium between the solution and the gas. By a modified type of calculation, the heat of solution determined included the latent heat of the evolved gas as well. The heat of solution thus calculated is greater than values obtained for CaCO_3_ by Wells and Taylor and by Bäckström in determinations in which only part of the CO_2_ went into solution. It is consistent with other thermochemical data in the literature.

## Figures and Tables

**Table 1:** Preparations of 3CaO·Al_2_O_3_·CaCO_3_·*n*H_2_O

Sample	1	2	3

Original concentration of mixture:			
CaO g/liter	1.129	1.158	1.473
Al_2_O_3_ g/liter	0.358	0.355	0.370
Na_2_CO_3_ g/liter	.342	.337	.386
Molar ratio:			
CaO: Al_2_O_3_	5.74	5.93	7.72
CO_2_: Al_2_O_3_	0.92	0.91	1.00
Final concentration of solution:			
CaO g/liter	.387	.414	0.653
Al_2_O_3_ g/liter	.007	.008	.001
Composition of product:			
Weight percent^a^			
CaO	40.19	39.66	40.38
Al_2_O_3_	18.04	18.22	17.78
CO_2_	7.95	7.74	8.56
H_2_O	33.82	34.38	33.28
Molar ratio:			
CaO:Al_2_O_3_	4.05	3.96	4.13
CO_2_:Al_2_O_3_	1.02	0.98	1.12
H_2_O: Al_2_O_3_	10.61	10.68	10.59
Molar ratios of compounds present to Al_2_O_3_:			
3CaO·Al_2_O_3_·CaCO_3_·*n*H_2_O	1.00	0.96	1.00
where *n*=	10.58	10.88	10.58
CaCO_3_	0.02		0.12
3CaO·Al_2_O_3_·6H_2_O		0.04	
Ca(OH)_2_	.03		.01
CO_2_		.02	
Heat of solution of the sample:			
–Δ*H* (from table 4) cal/g	226.07	229.25	224.74
Standard deviation of average	0.21	0.54	0.18
Corrected Heat of Solution of the			
3CaO·Al_2_O_3_·CaCO_3_·*n*H_2_O, –ΔH cal/g	225.75	225.86	227.48
kcal/mole	126.62	127.89	127.59

a The CaO/Al_2_O_3_ ratios were determined on wet samples, and the CO_2_ and H_2_O were determined on samples conditioned over saturated MgCl_2_ solution. For this reason, the analyses total exactly 100 percent.

**Table 2:** Reactants

	3CaO·Al_2_O_3_·6H_2_O	CaCO_3_
	
Composition		
Weight percent:		
CaO	44.52	56.00
Al_2_O_3_	26.93	…………
CO_2_	0.11	43.93
H_2_O	27.88	…………
Molar ratio:		
CaO: Al_2_O_3_	3.007	…………
CO_2_: Al_2_O_3_	0.009	…………
H_2_O: Al_2_O_3_	5.859	…………
CaO:CO_2_	…………	1.00
Molar ratios of compounds:		
3CaO·Al_2_O_3_·5.859H_2_O	1.000	…………
CaCO_3_	0.007	…………
CO_2_	.002	…………
Heat of solution of the materials (from tables 4 and 5)		
*–*Δ*H* cal/g	369.73±0.33	83.61 ±0.51
Corrected heat of solution of the reactant		
*–*Δ*H* cal/g	370.35_1_±0.33	83.61±0.51
kcal/mole	139.16_3_±0.12	8.37±0.05

**Table 3a:** Calibration of calorimeter and 600 grams 2N *HCl*, used for determining the heat of solution of *3CaO·Al_2_O_3_·6H_2_O* and the samples of *3CaO·Al_2_O_3_·CaCO_3_·nH_2_O*

Run	Time	I	E	Q	Δ*R_c_*	Heat capacity

	*sec*	*amp*	*Volts*	*j*	*Ohm*	*j/ohm*
1	599.91	0.188960	17.2276	1952.903	0.079767	a24,482.5_9_
2	599.85	.188982	17.2293	1953.128	.079847	24,460.8_8_
3	600.15	.188959	17.2272	1953.629	.079779	24,488.0_1_
3	600.21	.201466	18.3474	2218.603	.090500	24, 514.9_4_
5	599.79	.196921	17.9510	2120.215	.086527	24, 503.5_1_
6	660.04	.192244	17.5261	2223.865	.090897	24,465.7_6_
7	659.95	.191276	17.4388	2201.345	.090058	24,443.6_4_

Mean heat capacity					j/ohm	24,479.9_0_±9.43
Mean heat capacity					cal/ohm	5,850.8_4_±2.25

a It is not the authors’ intention to imply that the heat-capacity values are either precise or accurate to the number of figures tabulated. These figures are carried through the calculations and the heat-of-solution values obtained from them in tables 4 and 5 are rounded off.

**Table 3b:** Calibration of calorimeter and 740 grams 2*N HCl*, used for determining heat of solution of *CaCO_3_*. Average heater resistance 69.0995 ohms

Run	Time	I	I^2^RT=Q	Δ*R_c_*	Heat capacity

	*sec*	*amp*	*j*	*Ohm*	*j/ohm*
1	720.24	0.239042	2843.810	0.099817	a28,490.2_4_
2	720.32	.236182	2776.476	.097375	28,513.2_3_
3	780.21	.233174	2931.208	.102977	28,464.6_9_
4	960.20	.232667	3591.751	.126155	28,470.9_3_
5	839.72	.232183	3128.025	.109968	28,444.8_7_

Mean heat capacity				j/ohm	28,476.7_9_±11.64
Mean heat capacity				cal/ohm	6,806.1_2_±2.78

a See footnote, table 3a.

**Table 4:** Heat-of-solution determinations. Heat capacity of calorimeter, 24,479.90 joules/ohm

	Run	Corrected rise	Sample weight	Heat of solution, uncorrected –Δ*H*	Correction for heat capacity of sample –Δ*H*	Heat of solution, corrected –Δ*H*

		*Ohm*	*g*	*j/g*	*j/g*	*j/g*
3CaO·Al_2_O_3_·CaCO_3_·*n*H_2_O Sample 1	1	0.038552	0.9976	946.02	0.00	946.02
	2	.039513	1.0206	947.75	+.13	947.88
	3	.019889	0.5158	943.93	−.38	943.55
	4	.021232	.5492	946.39	−.29	946.10

Mean, before correction for impurities					j/g	945.89±0.88
					cal/g	226.07±0.21

Sample 2	1	0.039698	1.0186	954.06	−0.79	953.27
	2	.039823	1.0196	956.12	−.13	955.99
	3	.039254	0.9940	966.73	−.08	966.65
	4	.020605	.5258	959.32	−.08	959.24
	5	.019594	.4994	960.47	+.21	960.68

Mean, before correction for impurities					j/g	959.17±2.26
					cal/g	229.25±0.54

Sample 3	1	0.038027	0.9909	939.45	−0.21	939.24
	2	.038923	1.0147	939.03	−.13	938.90
	3	.020396	0.5298	942.42	−.33	942.09
	4	.020225	.5256	941.98	−.88	941.10

Mean, before correction for impurities					j/g	940.33±0.75
					cal/g	224.74±0.18

3CaO·Al_2_O_3_·5.859H_2_O	1	0.019390	0.3057	1552.72	+0.92	1553.64
	2	.018995	.3001	1549.47	+.50	1549.97
	3	.019701	.3122	1544.77	+.46	1545.23
	4	.027583	.4384	1540.21	+.25	1540.46
	5	.016714	.2647	1545.74	+.33	1546.07
	6	.018847	.2980	1548.23	+.38	1548.61
	7	.016869	.2669	1547.21	+.25	1547.46
	8	.019520	.3085	1548.94	+.46	1549.40
	9	.023494	.3732	1541.08	+.46	1541.54

Mean, before correction for impurities					j/g	1546.93± 1.34
					cal/g	369.73±0.33

**Table 5:** Heat-of-solution determinations, *CaCO_3_* Heat capacity of calorimeter, without bulb and sample assembly, 28,476.79 j/ohm

Run	Sample weight	Water	Corrected rise	Blank correction	Net rise	Heat capacity corrections		Total heat capacity of calorimeter	Heat of solution –Δ*H*
						Sample + glass	Water		

	*g*	*g*	*Ohm*	*Ohm*	*Ohm*	*j/ohm*	*j/ohm*	*j/ohm*	*j/g*
1	0.0000	4.0286	0.000456	………	a0.0001132				
2	.0000	7.9910	.000906	………	a.0001134				
3	.2247	5.2820	.003334	0.000598	.002736	+80.79	+212.17	28,769.75	350.31
4	.2247	4.5413	.003230	.000515	.002715	+79.87	+182.42	28,739.08	347.25
5	.2110	7.8531	.003486	.000890	.002596	+79.83	+315.43	28, 872.05	355.22
6	.2068	6.0117	.003146	.000681	.002465	+78.58	+241.46	28,796.83	343.25
7	.2277	4.7903	.003339	.000543	.002796	+81.59	+192.42	28,750.80	353.04

Mean heat of solution								j/g	349.81±2.13
								cal/g	83.61±0.51

a Net rise, ohm, of blank per g water.

## Footnotes

1 Figures in brackets refer to the literature references at the end of this paper.

2 The estimates of standard deviation quoted in this paper represent the standard deviation of the average, using *n*−1 degrees of freedom.

## 5. Appendix. Calculation of the Impurities, H_2_O Content, and Heat of Solution of a Preparation of Calcium Aluminate Monocarbonate

### Sample 1

From the mole ratios of the oxides (table 1), the mole ratio of 3CaO·Al_2_O_3_·CaCO_3_ to Al_2_O_3_ was taken as 1.00. This accounts for 4.00 moles of CaO and 1.00 mole of CO_2_, leaving a remainder of 0.05 mole of CaO and 0.02 mole of CO_2_. Since calcite was observed in the microscope in very small quantities, the remainder was calculated to 0.02 mole of CaCO_3_ and 0.03 mole of excess Ca(OH)_2_. The 0.03 mole of H_2_O corresponding to the Ca(OH)_2_ was deducted from the 10.61 moles of H_2_O, leaving 10.58 moles of H_2_O in the hydrated aluminate carbonate, or a composition of 3CaO·Al_2_O_3_·CaCO_3_·10.58H_2_O.

The heat of solution of each impurity was multiplied by its weight per mole of Al_2_O_3_ to obtain the calories produced by the impurity. The sum of the heats of solution of all the impurities was subtracted from the total heat (heat of solution of the sample multiplied by its total weight per mole of Al_2_O_3_) to obtain the heat effect of the pure aluminate carbonate. This result, divided by the mole ratio of pure compound to Al_2_O_3_ (in this case 1.00) is the corrected heat of solution in calories per mole. If this quotient is further divided by the formula weight of the pure compound, the heat of solution is obtained in calories per gram. Thus, for sample 1:

1.00 mole 3CaO·Al_2_O_3_·CaCO_3_·10.58H_2_O×560.90	= 560.90 g
0.02 mole CaCO_3_ ×100.09	= 2.00 g
0.03 mole Ca(OH)_2_ ×74.096	= 2.22 g
Total weight of sample per mole Al_2_O_3_	565.12 g
Total heat of solution per mole Al_2_O_3_=226.07 cal/g×565.12	= 127,757 cal.
—Heat of solution of CaCO_3_=83.61×2.00	= −167 cal
—Heat of solution of Ca(OH)_2_=436.41×2.22	= −969 cal
Net heat of solution of aluminate carbonate	= 126,621 cal
Heat of solution of aluminate carbonate	= 126.62 kcal/mole
	$=\frac{126,621}{560.90}=225.75\;\text{cal}/g$

The calculation in the case of sample 2 was based on the presence of 3CaO·Al_2_O_3_·6H_2_O and CO_2_ as impurities. A small quantity of the tricalcium aluminate hexahydrate was observed under the microscope.
